# Immunoexpression of Programmed Death-1 Receptor (PD-1) and Programmed Death-Ligand 1 (PD-L1) in Non-Small-Cell Lung Carcinoma and Its Correlation With Other Clinicopathological Parameters: A Cross-Sectional Study From North India

**DOI:** 10.7759/cureus.25243

**Published:** 2022-05-23

**Authors:** Michael L Anthony, Nilotpal Chowdhury, Mayank Mishra, Sudheer Tale, Kunnumbrath Arathi, Shalinee Rao

**Affiliations:** 1 Pathology, All India Institute of Medical Sciences (AIIMS) Mangalagiri, Mangalagiri, IND; 2 Pathology, All India Institute of Medical Sciences (AIIMS) Rishikesh, Rishikesh, IND; 3 Pulmonary Medicine, All India Institute of Medical Sciences (AIIMS) Rishikesh, Rishikesh, IND; 4 Pulmonary Medicine, Fortis Healthcare, Vijayawada, IND; 5 Pathology, Employees' State Insurance Corporation (ESIC) Medical College and Post Graduate Institutes for Medical Sciences and Research (PGIMSR), Chennai, IND

**Keywords:** tumor-infiltrating lymphocytes, non-small cell lung carcinoma, immunotherapy, pd-l1, pd-1

## Abstract

Aim

To study the prevalence of programmed death-1 receptor (PD-1) and programmed death-ligand 1 (PD-L1) positive cases in non-small-cell lung carcinoma (NSCLC) and their association with other clinicopathological parameters in a tertiary care setting in North India.

Material and methods

One hundred histologically proven NSCLC cases having sufficient tumor material from July 2016 to July 2018 were examined, and the prevalence of PD-1 and PD-L1 positivity in NSCLC was studied. In addition, H&E-stained sections were reviewed, and 100 consecutive cases meeting study criteria were identified as study cases. Histopathological categorization was done using a panel of immunohistochemical markers.

Statistical analysis and results

The PD-1 positivity in lymphocytes was 29% (95% CI: 20.4%-38.9%). Membranous positivity for PD-L1 in tumor cells was 27% (95% CI: 18.6%-36.8%) and in tumor-infiltrating lymphocytes was 22% (95% CI: 14.3%-31.4%). There was no statistically significant association between PD-1 or PD-L1 status with age, gender, smoking, pleural effusion, clinical stage, histological type, or lymphocyte infiltration.

Conclusion

The moderately high prevalence may justify routine testing for PD-1 or PD-L1 in NSCLC, which should preferably be carried out in all cases rather than any selected subsets. However, there was no significant correlation between PD-1 and PD-L1 with the clinical parameters studied.

## Introduction

Lung cancer remains one of the most lethal and most common cancers worldwide and in India. ​In both sexes combined, lung cancer is the most commonly diagnosed cancer [1]. ​Non-small-cell lung cancer (NSCLC) accounts for 75% of all lung cancers, including different subtypes, underlying relevant biological differences, and pathways [2]. Immunotherapy has been shown to significantly improve the overall survival and outcome of NSCLC patients [3,4]. ​Monoclonal antibodies targeting the programmed death-1 receptor (PD-1) (nivolumab [Opdivo] and pembrolizumab [Keytruda]) and programmed death-ligand 1 (PD-L1) (atezolizumab [Tecentriq]) are FDA-approved for use in NSCLC [4-7]. National Comprehensive Cancer Network guidelines currently recommend standard PD-L1 immunohistochemistry (IHC) testing in all advanced (stage IIIB and IV) lung squamous cell carcinomas and adenocarcinomas for selection of frontline pembrolizumab therapy [8]. Before being accepted in any country such as India, the validity of such guidelines needs examination. The prevalence of PD-1 and PD-L1 positivity in NSCLC needs to be established in any population before generalized guidelines are followed. However, such studies in India are scarce. Therefore, this study was designed to study the prevalence of PD-1 and PD-L1 in NSCLC in a tertiary care center in North India. Establishing the prevalence would give robustness for making any local guidelines for routine testing. Additionally, we also studied the relationship of PD-1 and PD-L1 status with other clinically relevant factors to understand the tumor biology of PD-1 and PD-L1 positive and negative tumors.

## Materials and methods

This was an observational cross-sectional study carried out in the ​Department of Pathology at a tertiary care hospital in North India. Core biopsy samples from 100 consecutive, histologically proven NSCLC cases of either sex having sufficient tumor material from July 2016 t July 2018 were included in this study. Clinical details were noted from case files and the Department of Radiology. Patients who had undergone chemotherapy or radiotherapy before core biopsy or had inadequate tissue for making sufficient stained slides were excluded from the study. Approval of this study was obtained from the Institutional Ethics Committee, AIIMS, Rishikesh (IRB No. #77/IEC/PGM/2016) before starting the study using formalin-fixed paraffin-embedded tissue samples. The cores were immediately put in 10% formalin and were fixed for six to forty-eight hours before processing. Due to the retrospective, anonymized character of the analysis on archival tissue embedded in paraffin blocks, written consent was not required.

Clinical TNM staging was done based on Union for International Cancer Control (UICC)/American Joint Committee on Cancer (AJCC) 8​th edition 2017 recommendations. H&E-stained sections were reviewed, and 100 consecutive cases meeting study criteria were identified as study cases. Histopathological typing was done using a panel of IHC markers (TTF-1, Napsin-A, p63, p40, Synaptophysin, and Chromogranin) from core biopsy tissue of cases diagnosed as lung carcinoma. H&E-stained sections of study cases were observed for lymphocyte load in reference to tumor tissue present and necrosis. Study cases were further subjected to IHC with PD-1 and PD-L1. Four-micron thick paraffin-embedded tissue sections were subjected to IHC with the following primary antibodies (pre-diluted) on positively charged slides: (i) PD-1 (Clone: EP239; Isotype: Rabbit IgG, PathnSitu); (ii) PD-L1 (Clone: CAL10; Isotype: Rabbit IgG, PathnSitu); (iii) p63 (Clone: 4A4; Isotype: Mouse IgG2α/, PathnSitu); (iv) p40 (Clone: ZR8; Isotype: Rabbit IgG, Master Diagnostica); (v) TTF-1 (Clone: EP229; Isotype: Rabbit IgG, PathnSitu); and (vi) Napsin A (Clone: EP205; Isotype: Rabbit IgG, PathnSitu).

Sections were examined under low power (10X) and high power (20X and 40X) fields to observe immunoreactivity. Proper controls were used to verify the staining characteristic of the IHC stained slides. Two Pathologists independently scored samples, and discrepancies were resolved by consensus. For PD-1 IHC, membrane or cytoplasm positivity in ≥5% lymphocytes were classified as positive [9]. For PD-L1, two types of positivity, lymphocyte and tumor cell membrane, were evaluated. At least 1% of lymphocytes showing cytoplasmic or membrane positivity for PD-L1 were classified as PD-L1 lymphocyte positive cases. At least 1% of tumor cells that showed complete or partial membranous staining for PD-L1 were classified as PD-L1 membrane positive cases. We used a semi-quantitative scoring for evaluating PD-1 and PD-L1 status, ranging from 0 to 3, representing negative, weakly positive, moderately positive, and strongly positive staining, respectively [9-10].​

The prevalence of PD-1 and PD-L1 (tumor cell membrane positive, lymphocyte positive) cases were calculated along with the exact 95% CIs. In addition, the association of PD-1 and PD-L1 positivity with the histological type, gender, smoking status, presence of pleural effusion, clinical stage, and percentage infiltration by lymphocytes were also studied. Fisher's exact test was used for each comparison, but for association with age and percentage infiltration by lymphocytes Mann-Whitney U test was used. The R Statistical environment (R Foundation of Statistical Computing, Vienna, Austria, 2014) was used for statistical analysis. This was a time-bound study; however, the sample size of 100 cases is sufficient to provide a CI width of ±0.1 at any prevalence.

## Results

**Figure 1:**
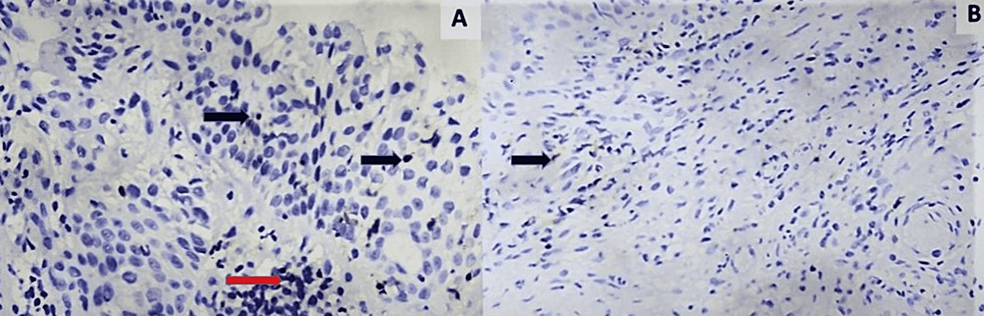
PD-1 positivity in lymphocytes. (A): Section shows a tiny aggregate of PD-1 positive lymphocytes (red arrow) in a case of squamous cell carcinoma. Also, note the PD-1 positive lymphocytes (black arrows) infiltrating the overlying dysplastic squamous epithelium (IHC X400). (B): PD-1 positive lymphocytes (black arrow) are seen scattered in the stroma amidst tumor cells (IHC X100). PD-1: Programmed death-1 receptor; IHC: Immunohistochemistry.

**Figure 2:**
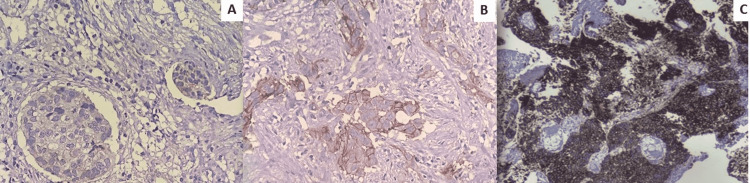
Membranous PD-L1 positivity in tumor cells. (A): 1+ (mild) positivity in tumor cells (IHC X400). (B): 2+ (moderate) positivity in tumor cells (IHC X400). (C): 3+ (strong) positivity in tumor cells (IHC X100). PD-L1: Programmed death-ligand 1; IHC: Immunohistochemistry.

**Figure 3:**
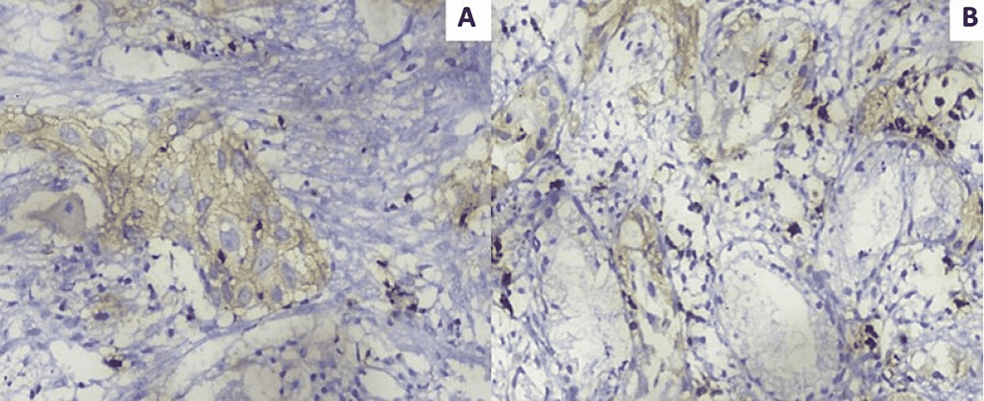
PD-L1 positivity in lymphocytes (IHC X400). (A) Tumor cells showing membranous positivity for PD-L1. Also seen are tumor-infiltrating lymphocytes, which are positive for PD-L1. (B) PD-L1 positive lymphocytes were seen scattered in the surrounding stroma. PD-L1: Programmed death-ligand 1; IHC: Immunohistochemistry.

Twenty-nine cases (29%, 95% CI = 20.4%-38.9%) out of hundred cases were found positive for PD-1 immunoexpression in lymphocytes. PD-1 positive lymphocytes were observed within the epithelial tumor cell formations and within the tumor stroma (Figure 1). Twenty-seven cases (27%, 95% CI =18.6%-36.8%) showed membranous positivity for PD-L1 (Figure 2). In twenty-two cases (22%, 95% CI = 14.3%-31.4%), tumor-infiltrating lymphocytes were positive for PD-L1, of which fifteen also showed tumor cell positivity (Figure 3). There was a significant association between PD-L1 cell positivity and lymphocyte positivity (P-value <0.001 by the Fisher's exact test).

No statistically significant association was found between PD-1 or PD-L1 status with age, gender, smoking status, pleural effusion, clinical stage, histological type, or percent infiltration by lymphocytes. Association P-values are given in Tables 1 and 2. There was also no significant association between PD-1 expression and either tumor cell PD-L1 expression or lymphocytic PD-L1 expression (p-values = 0.14 and 0.19, respectively).

**Table 1:** Showing the distribution of PD-1 and PD-L1 in tumor cells and PD-L1 in lymphocytes among the various pathological and clinical groups of non-small-cell lung cancer patients in the present study. In addition, the p-values for the association by the Fisher's Exact Test of the groups with PD-1 and PD-L1 status are also given. PD-1: Programmed death-1 receptor; PD-L1: Programmed death-ligand 1; NSCLC: Non-small-cell lung cancer.

Parameter		Number	PD-1		PD-L1 in tumor cell		PD-L1 in lymphocyte	
			Number	P-value	Number	P-value	Number	P-value
Tumor histology	Squamous cell carcinoma	78	22	0.793	19	0.367	18	1
	Adenocarcinoma	20	7		7		4	
	NSCLC (untyped)	2	0		1		0	
Gender	Males	88	23	0.101	24	1	19	0.723
	Females	12	6		3		3	
Smoking status	Yes	90	27	0.719	25	0.725	20	1
	No	10	2		2		2	
Clinical stage	Stage I-II	10	2	0.71	2	0.689	0	0.426
	Stage III-IV	90	27		25		22	
Pleural effusion	Present	39	10	0.654	9	0.645	7	0.47
	Absent	61	19		18		15	

**Table 2:** Showing the relationship of PD-1 and PD-L1 in tumor cells, and PD-L1 in lymphocytes status with age and percentage of lymphocytes in the tumor. The Mann-Whitney test calculates the statistical significance of difference. PD-1: Programmed death-1 receptor; PD-L1: Programmed death-ligand 1.

Immunopositivity	Age (years)		Percentage of ymphocytes	
	Median (Range)	P-value	Median (Range)	P-value
PD-1 Negative	60 (40-80)	0.83	10 (2-30)	0.04
PD-1 Positive	60 (35-90)		10 (5-25)	
PD-L1 in tumor cell Negative	60 (35-90)	0.35	10 (2-30)	0.83
PD-L1 in tumor cell Positive	60 (39-72)		10 (5-20)	
PD-L1 in lymphocyte Negative	60 (35-82)	0.81	10 (2-30)	0.40
PD-L1 in lymphocyte Positive	60 (41-90)		10 (5-20)	

## Discussion

**Figure 4:**
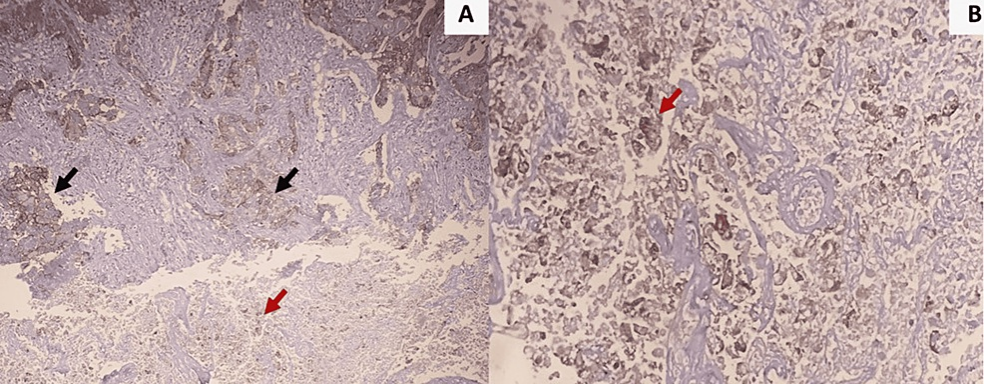
(A & B) Sections show tumors with extensive areas of necrosis. IHC for PD-L1 shows crisp membranous positivity in the tumor cells in the viable areas (black arrows in A, IHC X100). However, the necrotic foci show diffuse positivity for PD-L1, the intensity, and percentage of which are difficult to assess (red arrows in A and B). (B) High power view showing tumor cells with diffuse PD-L1 positivity (red arrows), entrapped in the necrotic debris (IHC X400). PD-L1: Programmed death-ligand 1; IHC: Immunohistochemistry.

PD-L1 immunoexpression was seen in the tumor cell membrane and tumor-infiltrating lymphocytes. Most targeted therapies require 1% membranous positivity for classifying a tumor as PD-L1 positive. Additionally, atezolizumab requires staining of 1% immune cells as well [4,11-12]. Vallonthaiel AG et al. considered membranous (complete circumferential or partial linear plasma membrane) and cytoplasmic staining at any intensity in >5% tumor cells or immune cells (IC) as positive for PD-L1 [13,14]. Our study considered PD-L1 positive even if 1% of the tumor cells showed membranous positivity or IC positivity. Tumor necrosis posed a problem for the identification of expression of PD-L1 in tumor cells. Therefore, it is important to select sections with minimal necrosis to reduce problems in interpretation (Figure 4). To identify cases of 1+ staining intensity, high power viewing was more useful compared to viewing at lower magnification.

The findings concerning PD-L1 correspond well with other published studies, which report IHC expression rates of 13-70% for PD-L1 in tumors of NSCLC patients [14-16]. Differences might be due to variabilities of the tumor microenvironment and to non-static expression at a single point in time [17]. We used a clone for PD-1 and PD-L1, which is for research use only and must be validated as a laboratory-developed test to be put into clinical use. The difference in staining characteristics between clones used to develop the antibodies may also account for some differences; however, these differences are likely to be mild and clinically non-significant for research in finding the prevalence. PD-1 immunoexpression was present in 29 cases (29%). This tallies well with other studies, which have found 35.2% to 22% positivity for PD-1 [9-10]. These PD-1 positive lymphocytes were observed within the epithelial tumor cell formations and within the tumor stroma. Additionally, PD-1 and PD-L1 expressions were found to be independent of one another. The clinical significance of this is unclear. Around 10% of PD-L1 negative NSCLC patients respond to targeted immune therapy; a subset of the PD-1 positive but PD-L1 negative cases may serve as potential candidates for immune therapy in clinical trials in search of finding these PD-L1 negative therapy responders.

We did not find a statistically significant relationship between PD-1 status, PD-L1 status, and other clinicopathological parameters (age, gender, histological type, smoking status, pleural effusion, and clinical stage). While previous studies have found a relationship between the tumor type and PD-1 or PD-L1 status, these studies have been inconsistent as to the direction of the relationship, with some studies finding a higher PD-L1 expression in squamous cell carcinoma and others noting higher PD-L1 expression in adenocarcinoma [9-10, 18-19]. D'Incecco A et al. found PD-1 expression to be associated with smoking history, whereas others have found that a history of smoking was associated with higher levels of PD-L1 expression [10, 20-23]. Takada K et al. have also found that the male gender and an advanced clinical stage are associated with PD-L1 expression [23]. Many other studies have found no such relationships. This finding implies that any NSCLC patient should be tested for PD-1and PD-L1 irrespective of the clinicopathological characteristics.

Fifteen out of twenty-seven cases showing membranous positivity for PD-L1 showed expression of PD-L1 in lymphocytes. These cases may represent cases with adaptive immune resistance, which would be most amenable to PD-L1 therapy. Seven cases were found to be PD-L1 positive in the ICs but negative in the tumor cells; these are thought to represent immune tolerance and immune system suppression by pathways other than the PD-1 axis [11]. This subset thus forms good potential candidates for trials for therapies affecting other immune targets.

Inter-assay variability in IHC staining is a potential limitation of the study. Mild discordance (up to 10%) between different assays has been demonstrated [24-25]. Therefore, for the purpose of treatment, approved PD-L1 assays or a validated alternative are recommended. The assay used in the present study was not meant for treatment purposes but meant for research use only. Therefore, there may be a mild variation in the prevalence of PD-L1 and PD-1 positivity if assayed by some other assay. We believe that such variations would be mild and will not change the primary conclusion of a moderately high prevalence of PD-1 and PD-L1 positivity among NSCLC patients in our population. Also, the study was undertaken in a tertiary care setting, raising the possibility of a selection bias. However, most cases of suspected NSCLC are serious enough to warrant referral to a tertiary care setting. Also, there was no statistically evident difference in PD-1 and PD-L1 positivity between different clinical subgroups. These factors possibly attenuate the risk of a markedly different prevalence of PD-1 and PD-L1 positivity in NSCLC among primary and secondary care patients of NSCLC at the geographical location of the study.

## Conclusions

The salient features of our study are that the data on the expression of PD-1 and PD-L1 in NSCLC, especially from India, is very limited. This is a study from India showing the expression of both these markers in NSCLC patients. The moderately high prevalence of PD-L1 and PD-1 in NSCLC in the studied population suggests that routine testing for PD-1 and PD-L1 may be justified, given the therapeutic potential of immunotherapy. The non-significance of the association between PD-L1 and PD-1 positivity with known clinicopathological parameters in NSCLC suggests that such routine testing should be carried out in all cases of NSCLC rather than in any selected subsets. Since this study was from a single center, the findings need to be corroborated for all stages of NSCLC from the different regions of the country for generalization. A multi-center study with more cases will be helpful in such generalizability.
